# Clinical Outcomes of N‐HA/pa66 and Titanium Mesh in the Treatment of Lower Cervical Spine Fractures and Dislocations During an 8‐Year Follow‐Up Period

**DOI:** 10.1111/os.70048

**Published:** 2025-05-01

**Authors:** Chen Wang, Yujie Hu, Ying Liu, Jiangbin Tang, Xi Yang

**Affiliations:** ^1^ Department of Orthopaedic Surgery West China Xiamen Hospital, Sichuan University Xiamen China; ^2^ State Key Laboratory of Oral Diseases and National Clinical Research Center for Oral Diseases West China Hospital of Stomatology, Sichuan University Chengdu China; ^3^ Department of Orthopaedic Surgery West China Hospital, Sichuan University Chengdu China

**Keywords:** anterior cervical corpectomy and fusion, lower cervical spine fracture and dislocation, nano‐hydroxyapatite/Polyamide66 cage, titanium mesh cage

## Abstract

**Objective:**

Studies evaluating the long‐term outcomes of the nano‐hydroxyapatite/polyamide 66 cages (n‐HA/PA66) in treating lower cervical spine fractures have not been reported. The objective is to compare the long‐term clinical and radiographic outcomes of titanium mesh cage (TMC) and‐HA/PA66 for anterior cervical corpectomy and fusion (ACCF) in the treatment of lower cervical spine fractures and dislocations.

**Method:**

This retrospective analysis included 223 patients treated at our hospital between January 2010 and January 2016 who had undergone single‐level anterior corpectomy for lower cervical spine fractures and dislocations (with a minimum follow‐up of 8 years) using either a TMC (*n* = 130) or an n‐HA/PA66 cage (*n* = 93). The radiographic parameters, including segmental alignment (SA), cage subsidence, plate‐to‐disc distance, cervical lordosis (CL), intervertebral height, and fusion status, along with clinical metrics such as Japanese Orthopedic Association (JOA) scores and visual analog scale (VAS) assessments, were systematically analyzed at preoperative, postoperative, and final follow‐up intervals for the patients involved in the study. The Chi‐Square (*χ* ^2^) test for categorical variables and the Student's *t*‐test for numerical data were used to assess differences between the two groups.

**Result:**

The mean follow‐up durations for the TMC group and n‐HA/PA66 group were9.81 ± 2.21 and 9.43 ± 0.92 years, respectively. Moreover, final fusion rates were not significantly different between the n‐HA/PA66 group and the TMC group (97.8% and 96.9%, respectively). The final cage subsidence was significantly lower in the n‐HA/PA66 group (1.56 ± 0.88 mm, with 17.6% subsidence of > 3 mm) than in the TMC group (2.70 ± 2.02 mm, with 36.9% subsidence) (*p* < 0.01). Furthermore, CL, SA, plate‐to‐disc distance, JOA scores, and VAS scores were not significantly different between the two groups (all *p* > 0.05).

**Conclusion:**

Within 8 years following single level ACCF surgery, the n‐HA/PA66 cage may be better than TMC in anterior cervical construction for treating lower cervical fractures and dislocations.

## Graphical abstract

To compare the long‐term clinical and radiographic outcomes of the TMC and the n‐HA/PA66 cage for anterior cervical corpectomy and fusion in the treatment of lower cervical spine fractures and dislocations. The n‐HA/PA66 cage may be superior to TMC in anterior cervical construction within 8 years after the operation.

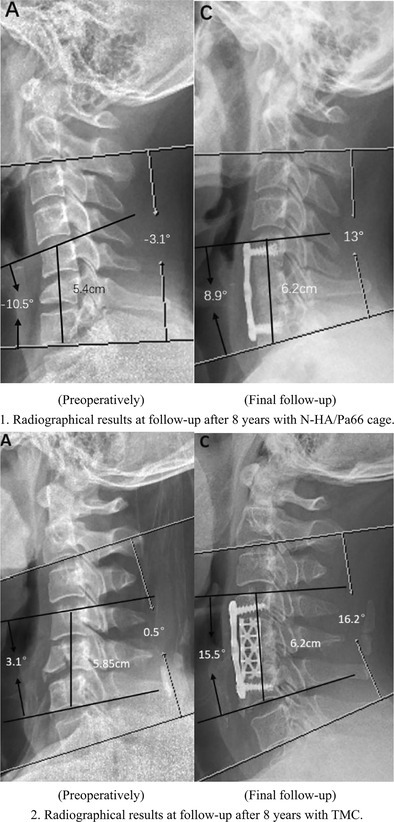

**Abbreviations**
3D‐CT: three‐dimensional computed tomography
ACCF: anterior cervical corpectomy and fusion
ASIA: American Spinal Injury Association
BMD: bone mineral density
BMI: body mass index
CL: cervical lordosis
ICC: intraclass correlation coefficients
IH: intervertebral height
JOA: Japanese Orthopedic Association
n‐HA/PA66: nano‐hydroxyapatite/polyamide66
PDD: plate‐to‐disc distance
SA: segmental alignment
TMC: titanium mesh cage
VAS: visual analog scale

## 1 Introduction

The majority of lower cervical spine fractures are brought on by high‐energy trauma, like falls and traffic accidents. The main types of injury are vertebral burst fracture or compression fracture [1]. These fractures often result in instability of the associated vertebral body and spinal cord injury, which affects the neurological function of patients. These fractures can even cause paraplegia. This has a significant effect on patients' quality of life. Lower cervical spine fractures are commonly treated by anterior cervical corpectomy and fusion (ACCF). ACCF is conducted via an anterior cervical approach, providing direct exposure to the compressed lesion and complete decompression of the spinal canal, thus alleviating spinal nerve compression and restoring spinal cord and nerve function [2].

Several studies have identified various supporting materials for ACCF, including autologous bone (iliac crest, fibula), allogeneic bone, and titanium mesh cage (TMC). But every material has advantages and disadvantages of its own [3, 4]. Because of its distinct benefits, including a high fusion rate, strong biocompatibility, and biomechanical qualities, autogenous iliac bone grafting is regarded as the gold standard for spinal fusion [5, 6]. Notably, TMC is widely used in clinical practice because it avoids bone removal from the body and addresses corresponding complications of the bone area. Besides, TMC is associated with a high decompression rate and high bone graft fusion rate, achieving good stability immediately [7, 8]. Nonetheless, TMC is associated with radiolucent opacity, increased stress shielding effect, and cage subsidence, limiting its application. Severe cage subsidence may lead to neck and shoulder pain, postoperative neurological deterioration, implant displacement or failure, and secondary surgery may be needed in severe cases [9, 10, 11]. A hollow bullet structure made of a composite of nano‐hydroxyapatite (n‐HA) and polyamide‐66 (PA66), the n‐HA/PA66 cage offers adequate mechanical support, high fusion rates, and low subsidence rates in anterior reconstruction because of its stiffness and elastic modulus, which are comparable to those of natural bone [12, 13]. Besides, the n‐HA/PA66 interbody fusion device has satisfactory long‐term clinical efficacy in treating thoracolumbar burst fractures and cervical spondylosis [14, 15]. Additionally, while treating cervical spondylosis, N‐HA/pa66 has a lower subsidence rate and a higher fusion rate than TMC [14, 16]. Comparing the long‐term clinical results of treating lower cervical spine fractures with TMC and n‐HA/PA66 cages hasn't been thoroughly studied. The purpose of the study should be itemized as follows: (i) To compare the long‐term clinical outcomes (e.g., neurological recovery, pain) between TMC and n‐HA/PA66 cages in the treatment of single‐level lower cervical spine fractures. (ii) To evaluate and contrast the radiological outcomes (e.g., fusion rates, cage subsidence rates, spinal stability, plate, and the adjacent disc space) of these two materials over a minimum follow‐up period of 8 years.

## 2 Methods

**FIGURE 1:**
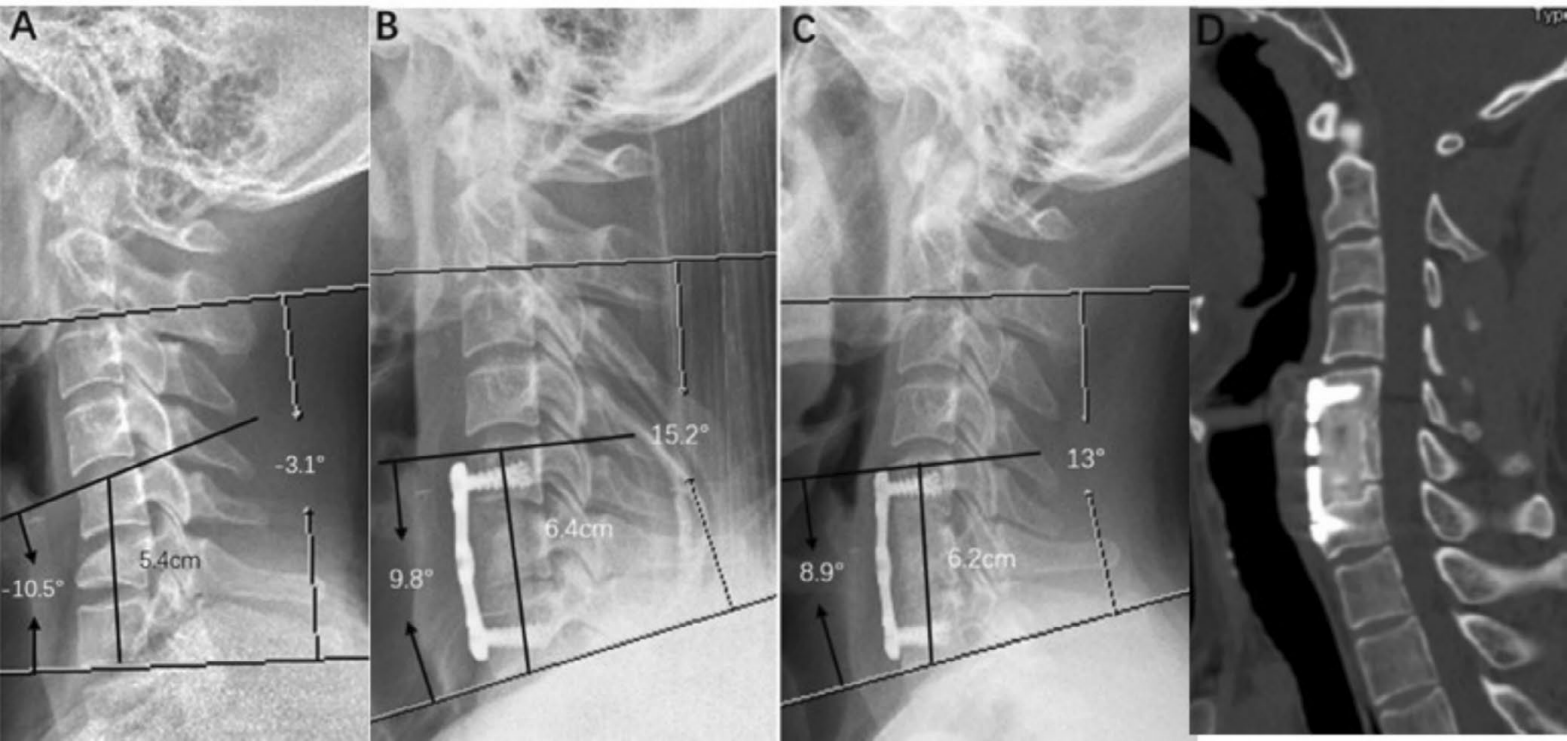
A 40‐year‐old man who underwent 1‐level corpectomy with a nano‐hydroxyapatite/polyamide66 strut for cervical reconstruction. The preoperative cervical X‐ray (A) (a significant collapse of the C6 vertebra): 1 week postoperatively lateral X‐ray (B) (C6 corpectomy and the n‐ha/pa66 cage used for reconstruction improved CL, SA, and IH). The radiographic films (C) and 3D‐CT (D) scan showing satisfying bony fusion with a little cage subsidence at the 9.5‐year follow‐up.

**FIGURE 2:**
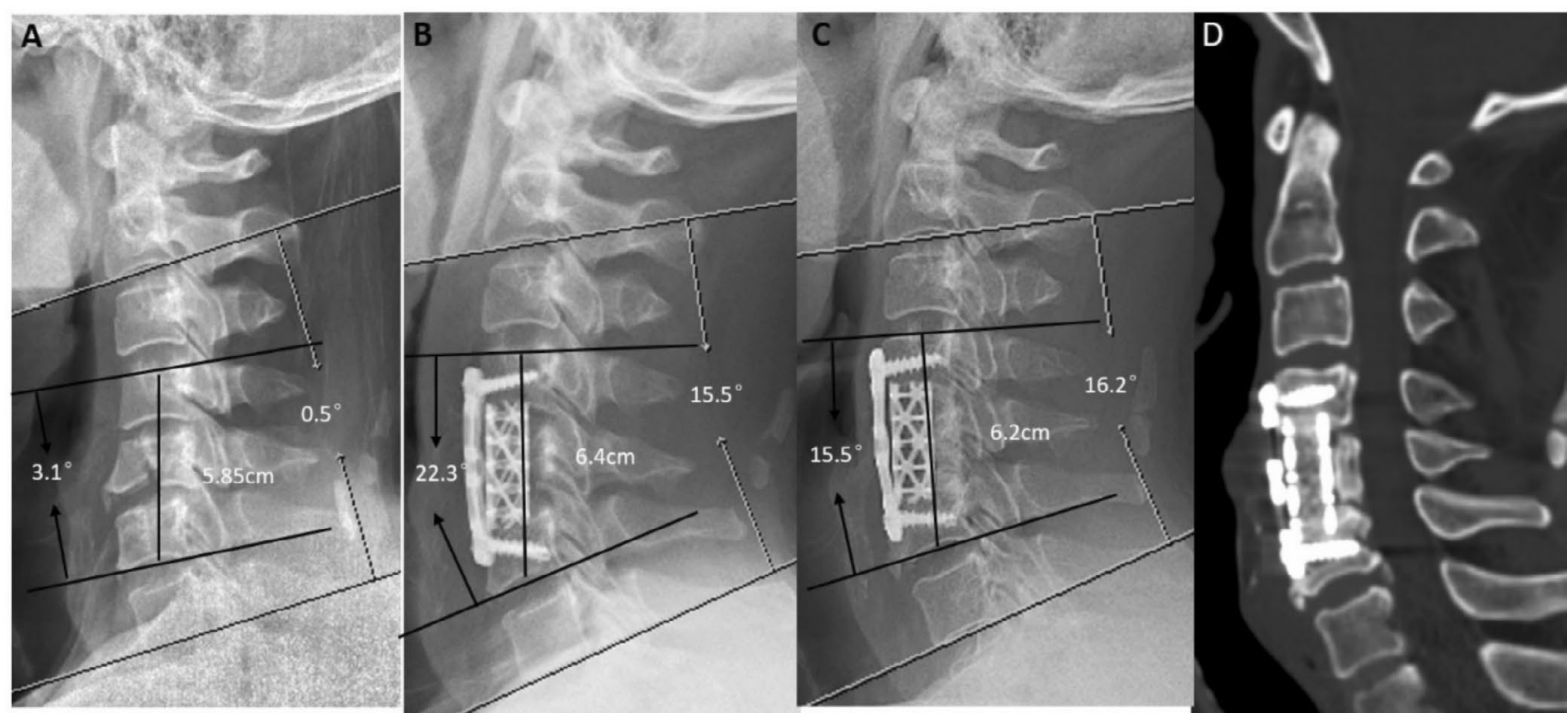
A 43‐year‐old male who underwent 1‐level corpectomy with a titanium mesh cage used for cervical reconstruction. The preoperative cervical X‐ray film (A) (showing a C5 burst fracture): 1 week postoperative lateral X‐ray (B) (C5 corpectomy and the titanium mesh cage used for reconstruction improved CL, SA, and IH). The radiographic films (C) and 3D‐CT (D) scan showing satisfying bony fusion at the 9‐year follow‐up with a little cage subsidence and partial bone resorption. CL, cervical lordosis; IH, intervertebral height; SA, segmental alignment.

Our institutional review board and local ethics committee approved this study, which complied with all applicable rules and regulations (Approval No. 2019‐654). An informed consent form was signed by each participant (Figures 1 and 2).

Patients with single level lower cervical spine fractures, with or without dislocation, resulting from acute trauma, who were admitted to our hospital between January 2010 and January 2016, were analyzed. Based on the American Spinal Injury Association (ASIA) classification of spinal cord injuries, we used the AO Spine subaxial cervical spine classification system to assess patients with lower cervical spine fractures; ACCF surgery was performed during their hospitalization. Inclusion criteria included: (1) patients whose single level lower cervical spine (C3‐7) fracture with or without dislocation was confirmed via comprehensive physical examination, X‐ray, CT, MRI, and other imaging examinations and met the surgical indications; (2) patients aged 18–75 years (both female and male); and (3) nonreoperation patients. Exclusion criteria were: (1) patients with minor compression fractures who did not meet the surgical indications; (2) Significant contraindications to surgery; (3) pathological fractures; (4) severe osteoporosis (*T*‐score < −2.5); and (5) patients with less than 8 years of postoperative follow‐up or those without full clinical follow‐up data. A total of 307 patients underwent ACCF surgery for lower cervical spine fractures, with 84 patients lost to follow‐up within 8 years postsurgery. Finally, 223 patients were included in the study (93 were treated with an n‐HA/PA66 cage (Sichuan National Nano Technology Co Ltd. Chengdu, SC) and 130 treated with a TMC (Medtronic SofamorDanek Inc. Memphis, TN)).

### 2.1 Radiographical Assessment

Preoperative, 1‐week postoperative, and final follow‐up imaging included frontal and lateral radiographs as well as three‐dimensional computed tomography (3D‐CT) of the cervical spine. The parameters derived from lateral radiography were also recorded cervical lordosis (CL), segmental alignment (SA), intervertebral height (IH), plate‐to‐disc distance (PDD) and cage subsidence (CS) were utilized to assess the radiological outcomes. The C2‐7 Cobb angle, also referred to as the CL angle, measures the Cobb angle between the upper and lower vertebrae at the implanted level, indicating segmental alignment. A positive angle signifies lordosis, whereas a negative angle denotes kyphosis. The cervical spine's immediate postoperative lateral radiograph was used to quantify the plate‐to‐disc distance (PDD), or the distance between the tips of the plate and the nearby cephalad disc. The distance between the midpoints of the superior endplate of the higher vertebra and the inferior endplate of the lower vertebra was used to calculate the intervertebral height of fusion. Subsidence was defined as the height difference between the fusion segment in the immediate postoperative period and subsequent follow‐ups. Cage subsidence of ≥ 3 mm was considered significant. Brantigan and Steffee's Grade 5 criteria—which classify grades 4 and 5 as fusion, Level 3 as inconclusive, and Grades 1 and 2 as unfused—were utilized to evaluate fusion status on 3D‐CT [17]. Three attending surgeons who were not on the original surgery team evaluated these radiological characteristics and quantified them using the mean of the three measurements. To evaluate the consistency of the data, intraclass correlation coefficients (ICC) were calculated for all radiographic parameters using a two‐way model and concordance type. The ICC values for CL, IH, SA, and PDD were found to be 0.902, 0.870, 0.910, and 0.834, respectively, indicating excellent to good reliability for all measurements.

### 2.2 Clinical Assessment

Preoperative clinical information, including gender, age, accompanied diseases, intraoperative blood loss, bone mineral density (BMD), BMI, follow‐up time, Operation time, and other general information, were obtained. The patients were evaluated neurologically using the American Spinal Injury Association (ASIA) impairment scale. Additionally, their scores were compared using the visual analog scale (VAS) and the Japanese Orthopedic Association (JOA) scale at preoperative, 1‐week postoperative, and final follow‐up assessments.

### 2.3 Statistical Analysis

SPSS software, version 26.0 (SPSS Inc., Chicago, IL, USA) was utilized for all statistical analyses. Parametric continuous variables were presented as means ± standard deviations. The Chi‐Square (*χ* ^2^) test for categorical variables and the Student's *t*‐test or Wilcoxon's rank sum test for numerical data were used to assess differences between the two groups. *p*‐Values less than 0.05 were regarded as statistically significant.

## 3 Results

This study included a total of 223 patients (180 males and 43 females) with an average age of 43.1 years (ranging from 18 to 81 years). The mean follow‐up duration was 9.57 years (ranging from 8 to 14 years). Patients were divided into two groups: the n‐HA/PA66 group (*n* = 93) and the TMC group (*n* = 130). Additionally, there were no significant differences between the two groups in terms of age, gender, BMI, comorbidities, fracture stage, and classification (*p* > 0.05). The demographic data and preoperative clinical characteristics are summarized in Table 1.

**TABLE 1:** Patient demographic data and clinical characteristics.

Variable	n‐HA/PA66 group (*n* = 93)	TMC group (*n* = 130)	*p*
Age (years)	42.34 ± 15.02	43.59 ± 13.93	0.52
Male (cases)	77	103	0.51
Follow‐up (years)	10.00 ± 2.01	9.81 ± 2.21	0.50
BMI (kg/m^2^)	22.46 ± 2.52	22.81 ± 2.50	0.31
Osteoporosis	5	8	0.81
Hypertension	12	20	0.60
Diabetes	9	15	0.66
Alcohol	16	16	0.30
Smoke	15	18	0.64
Injured site			
C3	2	5	0.93
C4	16	22	
C5	34	51	
C6	25	33	
C7	16	19	
ASIA in admission			
A	6	9	0.78
B	10	21	
C	26	38	
D	30	37	
E	21	25	

Abbreviations: ASIA, American Spinal Injury Association; BMI, body mass index; n‐HA/PA66, nano‐hydroxyapatite/polyamide66; TMC, titanium mesh cage.

### 3.1 Radiographical Outcome

The preoperative IH in the n‐HA/PA66 group and TMC group were 51.03 ± 5.30 mm and 50.37 ± 4.50 mm, respectively. IH increased to 57.13 ± 5.23 mm and 57.08 ± 3.03 mm in the n‐HA/PA66 and TMC groups, respectively, after surgery. Notably, IH significantly increased postoperative. However, The loss of interbody height was higher in the TMC group than in the n‐HA/PA66 group at the final follow‐up (1.56 ± 0.88 vs. 2.70 ± 2.02 mm; *p* < 0.01). Also, cage subsidence was markedly higher in the TMC group than in the n‐HA/PA66 group at the final follow‐up (17.6% vs. 36.9%; *p* < 0.01). But, there was no significant difference in IH, PDD, SA, and CL at any time after surgery. The fusion rate was slightly higher in the n‐HA/PA66 group than in the TMC group (97.8% vs. 96.9%; *p* = 0.67). (Table 2).

**TABLE 2:** Radiographic and clinical outcomes.

Variable	n‐HA/PA66 group (*n* = 70)	TMC group (*n* = 71)	*p*
CL (°)			
Preoperatively	7.4 ± 8.5	8.3 ± 7.7	0.43
1 week postoperatively	15.2 ± 4.6	14.4 ± 3.5	0.15
Final follow‐up	13.9 ± 4.1	13.7 ± 3.4	0.60
IH (mm)			
Preoperatively	51.0 ± 5.3	50.4 ± 4.5	0.32
1 week postoperatively	57.1 ± 5.2	57.1 ± 3.0	0.94
Final follow‐up	55.6 ± 4.8	54.5 ± 2.3	0.06
Subsidence(mm)	1.6 ± 0.9	2.7 ± 2.0	0.01
Final subsidence rate (%)	16/93 (17.2%)	48/130 (36.9%)	0.01
Fusion rate (%)	91/93 (97.8%)	126/130 (96.9%)	0.67
SA (°)			
Preoperatively	−6.7 ± 13.4	−7.5 ± 11.3	0.83
1 week postoperatively	8.6 ± 8.1	8.4 ± 7.7	0.10
Final follow‐up	6.5 ± 7.8	6.0 ± 6.6	0.82
PPD (mm)			
1 week postoperatively	4.8 ± 2.6	4.5 ± 2.5	0.72
Final follow‐up	3.8 ± 2.6	3.0 ± 2.5	0.34
VAS scores			
Preoperatively	6.72 ± 1.25	7.01 ± 1.07	0.07
1 week postoperatively	3.24 ± 0.91	3.38 ± 0.84	0.24
Final follow‐up	1.57 ± 1.30	1.78 ± 1.23	0.23
JOA scores			
Preoperatively	7.74 ± 3.81	8.12 ± 4.65	0.50
1 week postoperatively	9.52 ± 4.02	9.45 ± 4.47	0.07
Final follow‐up	10.97 ± 4.28	10.72 ± 4.22	0.66

Abbreviations: CL, cervical lordosis; IH, intervertebral height; JOA, Japanese Orthopedic Association; nano‐hydroxyapatite/polyamide 66; PPD, plate‐to‐disc distance; SA, segmental alignment; TMC, titanium mesh cage; VAS, visual analog scale.

### 3.2 Clinical Outcome

Operative timein the n‐HA/PA66 group and TMC group were (101.31 ± 7.97 vs. 99.96 ± 8.63 min; *p* = 0.27). Blood lossin the in the n‐HA/PA66 group and TMC group were (86.46 ± 8.23 vs. 88.19 ± 11.03 mL; *p* = 0.18). Surgery significantly improved the JOA and VAS scores in the two groups. The VAS scores and JOA at the last follow‐up were 1.57 ± 1.30 and 10.97 ± 4.28, respectively, for the n‐HA/PA66 group; and 1.78 ± 1.23 and 10.72 ± 4.22, respectively, for the TMC group (*p* > 0.05) (Table 2). 8 and 13 intraoperative cerebrospinal fluid (CSF) leak cases were reported in the n‐HA/PA66 group and the TMC group, respectively. CSF leak was caused by acute fracture‐dislocation, damaging the dura mater, and recovered following an extended period of postoperative pressured drainage. Besides, one case of superficial tissue infection was detected in both the n‐HA/PA66 group and the TMC group. The two patients were treated by changing the dressing on the surgical incision.

## 4 Discussion

### 4.1 Main Findings

The study investigated the long‐term clinical and radiological outcomes of single‐level ACCF in the treatment of lower cervical spine fractures and dislocations. Compared with TMC, the n‐HA/PA66 cage showed a lower subsidence rate.

### 4.2 Treatment of Cervical Spine Fracture‐Dislocation and Selection of Bone Graft Materials

Treatment of cervical fracture‐dislocation with spinal cord injury involves four key principles, including gentle reduction, complete decompression, reconstruction of cervical spinal lordosis, and rigid immobilization [18]. An efficient and dependable procedure for treating cervical fracture‐dislocation is ACCF. ACCF can achieve compressed lesion, complete decompression of the spinal canal, alleviate spinal nerve compression, and promote restoration of spinal cord and nerve function. The methods for decompressing the cervical spinal canal involve anterior, posterior, or combined anterior–posterior techniques, which can be performed in a single stage or in multiple stages [19, 20].

In addition, cervical reconstruction is an important procedure for cervical fracture‐dislocation treatment. Autogenous bone graft is usually obtained from the iliac or fibula. Autogenous bone transplantation has a high fusion rate and is the standard for bone material transplantation. However, autogenous bone transplantation is associated with blood loss, infection, hematoma, and pain in the donor site, limiting its application [21, 22]. Allogeneic bone transplantation has also been used for vertebral body reconstruction. Although this transplantation avoids complications at the donor site, allograft bone transplantation is associated with rejection, low fusion rate, and high collapse rate [23]. As a result, TMC has been developed and widely used for cervical fracture‐dislocation treatment. TMC is filled with an autograft from the vertebral body, providing early biomechanical stability to the anterior column, thereby restoring and maintaining the height of the intervertebral space and the cervical curvature. Nonetheless, because of its elastic modulus, TMC is linked to high cage subsidence rates, radiolucent opacity, and an enhanced stress shielding effect [9, 10, 24].

By incorporating nano‐HA into PA66, the n‐HA/PA66 composite is produced, simulating the distribution of apatite within a collagen matrix found in genuine bone. Consequently, the n‐HA/PA66 composite blends the elastic qualities of PA66 with the mechanical strength of HA. A previous study demonstrated that n‐HA is related to PA66 via hydrogen bonds through the formation of a carboxyl‐calcium‐carboxyl linkage [13, 25]. For many years, the n‐HA/PA66 composite has been utilized for spinal reconstruction. In a previous study, 35 patients who had single‐level cervical corpectomy and fusion employing n‐HA/PA66 cages saw a 97% fusion rate and a 6% subsidence rate [16]. Additionally, Yang et al. used n‐HA/PA66 for anterior cervical reconstruction to treat lower cervical fractures and dislocations, and they obtained a 4‐year fusion rate of 97.6% and a subsidence rate of 4% [26]. The n‐HA/PA66 group in this study had a little greater fusion rate (97.8% vs. 96.9%) than the TMC group. At an 8‐year follow‐up, Hu et al. [27] also found that the n‐HA/PA66 group had a greater fusion rate (96.4% vs. 94.2%) than the TMC group. These findings indicate that n‐HA/PA66 has a satisfactory fusion rate in cervical spine reconstruction, possibly due to the circular design and fenestrations of n‐HA/PA66, which increase the cross‐sectional area. This enhances stability and contact between the graft and vertebral bodies, facilitating fusion.

### 4.3 Comparison of Cage Subsidence Between n‐HA/PA66 and TMC

In this study, the final subsidence rate was lower in the n‐HA/PA66 group than in the TMC group (17.6% vs. 36.9%). The subsidence distance was also lower in the n‐HA/PA66 group than in the TMC group (1.56 ± 0.88 vs. 2.70 ± 2.02 mm). Various factors influence fusion device subsidence, including intraoperative endplate preparation, osteoporosis, and the properties and shape of the fusion device materials [28]. Zhang et al. [29] also found that the subsidence rate is markedly lower in the n‐HA/PA66 than in the TMC group at a 4‐year follow‐up (4% vs. 24%). Similarly, Hu et al. [27] showed that the subsidence rate is substantially lower in the n‐HA/PA66 group than in the TMC group at an 8‐year follow‐up (18.2% vs. 40.4%). In this study, the subsidence rate of the n‐HA/PA66 and TMC groups was lower than rates reported by Hu et al., possibly due to the young age of the included patients. Specifically, Hu et al. reported that the average age of patients in the n‐HA/PA66 group was 56.5 years (42.3 years in this study). Younger age indicates a lower prevalence of osteoporosis, significantly influencing subsidence [30].

Loss of intervertebral height, cervical kyphosis, foraminal stenosis, and recompression of the spinal cord and nerve roots might result from cage subsidence during follow‐up [24]. Severe cases may even require secondary surgery [31]. Subsidence is associated with high pressures transmitted through interbody spacers over a limited surface area. The n‐HA/PA66 cages exhibit a low Young's modulus, comparable to that of natural bone, which leads to reduced stress shielding. The Young's modulus of the n‐HA/PA66 cage (5.6 GPa) is significantly lower than that of TMC (110 GPa) [16, 32]. The elastic modulus of TMC is much higher than that of n‐HA/PA66, resulting in greater stress shielding and serious cage subsidence, explaining the higher subsidence in TMC. Moreover, Sharp imprints on the TMC are intended to prevent translation and secure the cage to the nearby endplates. But because the pointed footprints reduce the area of contact between the cage and the endplate, more cutting and penetration from the cage into the vertebra occurs [16]. The n‐HA/PA66 cage has a circular design and fenestration, which increase the cross‐sectional area, thus enhancing stability and promoting contact between the graft and vertebral bodies, facilitating fusion, as well as reducing the incidence and extent of cage subsidence.

### 4.4 Comparison of Radiographical and Clinical Outcomes

Studies have suggested that a shorter distance between the plate and the adjacent disc space increases the likelihood of developing ossification at adjacent levels [33, 34]. The N‐HA/PA66 group exhibited a smaller PDD than the TMC group at the final follow‐up (3.8 ± 2.6 mm vs. 3.0 ± 2.5 mm). Moreover, the cage subsidence directly reduced the plate and the adjacent disc space. Additionally, the degree of screw cutting was more severe in the TMC group than in the N‐HA/PA66 group, leading to a higher incidence of adjacent‐level ossification and adjacent segment disease compared with the N‐HA/PA66 group. At the last follow‐up, both groups demonstrated improvements in CL and SA compared to preoperative values, although no significant differences were observed between the two groups. Therefore, both n‐HA/PA66 and TMC cages can effectively restore the physiological curvature of the cervical spine. Additionally, there were no significant differences in VAS and JOA scores between the two cage types at any postoperative stage, with both achieving significant improvements compared to preoperative levels.

### 4.5 Strengths and Limitations

Strengths: (i) This study presents the first comparative analysis of long‐term clinical and radiographical outcomes in the treatment of lower cervical spine fractures using TMC and n‐HA/PA66 cages. Limitations: (i) This is a single‐center retrospective study with a limited sample size. (ii) Cage selection was not randomized, and physician‐related factors may have influenced the results, potentially introducing biases and confounding variables. Therefore, a prospective, randomized, multicenter study is needed to compare the long‐term effects of these two cages.

## 5 Conclusion

Compared with TMC, the n‐HA/PA66 cage showed a lower subsidence rate and higher radiolucency. These findings indicate that the n‐HA/PA66 cage is more suitable in the treatment of single‐level fracture and dislocation of the lower cervical spine than TMC.

## Author Contributions

Chen Wang and Yujie Hu co‐authored this article and contributed equally to this work. Xi Yang is responsible for the integrity of the study from inception to publication and should be considered the corresponding author. Chen Wang, Xi Yang, and Jiangbin Tang contributed to the concept and design of this study. Yujie Hu and Ying Liu were responsible for the literature search, data extraction, bias assessment, and data analysis.

## Conflicts of Interest

The authors declare no conflicts of interest.
